# Matrix metalloproteinases: expression and regulation in the endometrium during the estrous cycle and at the maternal-conceptus interface during pregnancy in pigs

**DOI:** 10.5713/ab.22.0478

**Published:** 2023-05-02

**Authors:** Inkyu Yoo, Soohyung Lee, Yugyeong Cheon, Hakhyun Ka

**Affiliations:** 1 Division of Biological Science and Technology, Yonsei University, Wonju 26493, Korea

**Keywords:** Endometrium, Matrix Metalloproteinase, Pig, Pregnancy

## Abstract

**Objective:**

Matrix metalloproteinases (MMPs) are a family of endoproteases produced by various tissues and cells and play important roles in angiogenesis, tissue repair, immune response, and endometrial remodeling. However, the expression and function of MMPs in the pig endometrium during the estrous cycle and pregnancy have not been fully elucidated. Thus, we determined the expression, localization, and regulation of *MMP2*, *MMP8*, *MMP9*, *MMP12*, and *MMP13* in the endometrium throughout the estrous cycle and at the maternal-conceptus interface during pregnancy in pigs.

**Methods:**

Endometrial tissues during the estrous cycle and pregnancy and conceptus and chorioallantoic tissues during pregnancy were obtained and the expression of MMPs was analyzed. The effects of steroid hormones and cytokines on the expression of MMPs were determined in endometrial explant cultures.

**Results:**

Expression levels of *MMP12* and *MMP13* changed during the estrous cycle, while expression of *MMP2*, *MMP9*, *MMP12*, and *MMP13* changed during pregnancy. Expression of *MMP2*, *MMP8*, and *MMP13* mRNAs was cell type-specific at the maternal-conceptus interface. Gelatin zymography showed that enzymatically active MMP2 was present in endometrial tissues. In endometrial explant cultures, estradiol-17β induced the expression of *MMP8* and *MMP12*, progesterone decreased the expression of *MMP12*, interleukin-1β increased the expression of *MMP2*, *MMP8*, *MMP9*, and *MMP13*, and interferon-γ increased the expression of *MMP2*.

**Conclusion:**

These results suggest that MMPs expressed in response to steroids and cytokines play an important role in the establishment and maintenance of pregnancy by regulating endometrial remodeling and processing bioactive molecules in pigs.

## INTRODUCTION

The endometrium undergoes different degrees of morphological and structural changes during the estrous cycle and pregnancy in a species-specific manner [1,2]. In primates, which have menstrual cycles and in which an invasive hemochorial placenta is formed, the changes in morphology are dramatic compared to other species, including pigs, in which a non-invasive epitheliochorial placenta is formed [3]. During pregnancy, extensive tissue remodeling in endometrial and placental tissues occurs to reduce the interhemal distance and increase utero-placental associations irrespective of placentation type [4]. The changes in the endometrium during the reproductive cycle, pregnancy, and parturition and in the placenta during pregnancy occur constantly and are accompanied by tissue remodeling via degradation of the extracellular matrix (ECM) [5,6]. Endometrial and/or placental tissue remodeling is involved in various critical reproductive processes, including menstruation, decidualization, trophoblast invasion, placental development, and spiral artery regeneration [5,6]. ECM degradation during endometrial and placental tissue remodeling is mediated primarily by matrix metalloproteinases (MMPs), which degrade most collagen fibers in the ECM and basement membrane and process a variety of bioactive molecules such as cytokines and chemokines [7]. Through this activity, MMPs also play important roles in the regulation of apoptosis, angiogenesis, tissue repair, and immune responses [8]. Because of the ability of MMPs to break down ECM physical barriers, abnormal expression of MMPs is associated with many diseases such as cancer, bone disease, and vascular disease [9–11].

MMPs belong to a family of zinc-dependent endoproteases comprising at least 28 members with significant sequence homology [12]. Members of the MMP family are commonly classified as collagenases, gelatinases, stromelysins, membrane-type MMPs, or others based on their domain organization and substrate preference [13]. MMPs can process bioactive molecules such as cytokines and chemokines thereby activating or inactivating them [14]. MMPs are produced by various tissues and cells, including connective tissue, fibroblasts, osteoblasts, endothelial cells, vascular smooth muscle cells, macrophages, neutrophils, lymphocytes, and cytotrophoblasts [8]. The expression of MMPs is induced by a variety of cytokines and growth factors, including interleukins (ILs), interferons (IFNs), epidermal growth factors (EGF), vascular endothelial growth factor (VEGF), tumor necrosis factor-α (TNF-α), and TNF-β [15].

In humans, the functional layer of the endometrium routinely exfoliates during menstruation, which is mediated by MMP2 and MMP14 [2,16]. During early pregnancy, MMP2 and MMP9 are involved in the implantation process via the degradation of collagen IV, which constitutes most of the maternal basement membrane [17]. In rhesus monkeys, MMP9 expressed in the endometrium and trophoblasts is involved in normal placentation [18]. Compared to primates and rodents, dramatic morphological changes of the endometrium do not occur in pigs during the estrous cycle or pregnancy as pigs undergo non-invasive implantation. However, transcriptomic studies have reported that several MMPs are differentially expressed in the endometrium during the peri-implantation period in pigs [19,20], suggesting that MMPs may play important roles in the establishment of pregnancy in pigs. Nevertheless, the expression and regulation of MMPs in the endometrium throughout the estrous cycle and during pregnancy have not been fully elucidated in pigs.

Therefore, to expand our understanding of the expression and function of MMPs in the endometrium during the estrous cycle and pregnancy in pigs, we evaluated: i) the expression of *MMP2*, *MMP8*, *MMP9*, *MMP12*, and *MMP13* in the endometrium during the estrous cycle and pregnancy and in conceptus tissues during the early pregnancy and chorioallantoic tissues during mid-to-late pregnancy; ii) the localization of *MMP2*, *MMP8*, and *MMP13* mRNAs in the endometrium during the estrous cycle and pregnancy; iii) the presence of active MMP2 enzyme in endometrial tissues; and iv) the regulation of *MMP2*,* MMP8*, *MMP9*, *MMP12*, and *MMP13* expression by steroid hormones and cytokines in endometrial explant tissues.

## MATERIALS AND METHODS

### Animals and tissue preparation

All experimental procedures involving animals were conducted in accordance with the Guide for Care and Use of Research Animals in Teaching and Research and approved by the Institutional Animal Care and Use Committee of Yonsei University (No. YWC-P120) and the National Institute of Animal Science (No. 2015-137). Sexually mature crossbred female gilts of similar age (6 to 8 months) and weight (100 to 120 kg) were assigned randomly to either cyclic or pregnant status. Gilts assigned to the pregnant uterus status group were artificially inseminated with fresh boar semen at the onset of estrus (day 0) and 12 h later. The reproductive tracts of gilts were obtained immediately after slaughter on either days 0 (onset of estrous behavior), 3, 6, 9, 12, 15, or 18 of the estrous cycle (21-day cycle: days 0 to 3, estrus; days 3 to 6, metestrus; days 6 to 15, diestrus; days 15 to 0, proestrus) and either days 10, 12, 15, 30, 60, 90, or 114 of pregnancy (n = 3 – 6/d/status). Pregnancy was confirmed by the presence of apparently normal spherical to filamentous conceptuses in uterine flushings on days 10, 12, and 15 and the presence of embryos and placenta on the later days of pregnancy. Uterine flushings were obtained by introducing and recovering 25 mL phosphate-buffered saline (PBS; pH 7.4) into each uterine horn. Chorioallantoic tissues were obtained from days 30, 60, 90, and 114 of pregnancy (n = 3 – 4/d). Endometrial tissues from prepubertal gilts (n = 8; approximately 6 months of age) that had not undergone the estrous cycle with no corpus luteum formed were obtained from a local slaughterhouse. Endometrial tissue, dissected free of the myometrium, was collected from the middle portion of each uterine horn, snap-frozen in liquid nitrogen, and stored at −80°C prior to RNA extraction. For *in situ* hybridization, cross-sections of endometrium were fixed in 4% paraformaldehyde in PBS (pH 7.4) for 24 h and then embedded in paraffin as described previously [21].

### Total RNA extraction and reverse transcription polymerase chain reaction for *MMP* cDNAs

Total RNA was extracted from endometrial, conceptus, and chorioallantoic tissues using TRIzol reagent (Invitrogen, Carlsbad, CA, USA) according to the manufacturer’s recommendations. The quantity of RNA was assessed spectrophotometrically, and the integrity of RNA was validated by electrophoresis in 1% agarose gels. Four micrograms of total RNA from endometrial, conceptus, and chorioallantoic tissues were treated with DNase I (Promega, Madison, WI, USA) and reverse transcribed using SuperScript II Reverse Transcriptase (Invitrogen, USA) to obtain complementary DNAs (cDNAs). cDNA templates were then diluted 1:4 with nuclease-free water and amplified by polymerase chain reaction (PCR) using Taq polymerase (Takara Bio, Shiga, Japan) and specific primers based on porcine *MMP2*, *MMP8*, *MMP9*, *MMP12*, and *MMP13* mRNA sequences. PCR conditions, sequences of primer pairs for *MMP2*, *MMP8*, *MMP9*, *MMP12*, and *MMP13*, and expected product sizes are listed in Table 1. PCR products were separated on 2% agarose gels and visualized by ethidium bromide staining. The identity of each amplified PCR product was verified by sequence analysis after cloning into the pCRII vector (Invitrogen, USA).

### Quantitative real-time reverse transcription polymerase chain reaction

To analyze expression of *MMP2*, *MMP8*, *MMP9*, *MMP12*, and MMP13 mRNAs in the endometrial and chorioallantoic tissues, real-time reverse transcription polymerase chain reaction (RT-PCR) was performed using the Applied Biosystems StepOnePlus System (Applied Biosystems, Foster City, CA, USA) and SYBR Green. Complementary DNAs were synthesized from 4 μg total RNA isolated from different uterine endometrial tissues, and newly synthesized cDNAs (total volume of 21 μL) were diluted 1:4 with nuclease-free water and then used for PCR. Power SYBR Green PCR Master Mix (Applied Biosystems, USA) was used for PCR reactions. The final reaction volume of 20 μL included 2 μL of cDNA, 10 μL of 2X Master mix, 2 μL of each primer (100 nM), and 4 μL of dH_2_O. PCR conditions and sequences of primer pairs are listed in Table 1. Results are reported as expression relative to the level detected on day 12 of the estrous cycle for endometrial tissues, day 30 of pregnancy for chorioallantoic tissues, or the control group in endometrial explant tissues after normalization of the transcript amount to the endogenous porcine ribosomal protein L7 (*RPL7*), ubiquitin B (*UBB*), and TATA-binding protein (*TBP*) controls by the 2^−ΔΔCT^ method [22].

### *In situ* hybridization

A nonradioactive *in situ* hybridization procedure was performed as described previously (Braissant and Wahli, 1998) with some modifications. Sections (5 μm thick) were rehydrated through successive baths in xylene, 100% ethanol, 95% ethanol, and diethylpyrocarbonate (DEPC)-treated water. Tissue sections were boiled in citrate buffer, pH 6.0, for 10 min. After washing in DEPC-treated PBS, they were digested using 5 μg/mL proteinase K (Sigma, St. Louis, MO, USA) in 100 mM Tris-HCl and 50 mM ethylenediaminetetraacetic acid (EDTA), pH 7.5, at 37°C. After postfixation in 4% paraformaldehyde, tissue sections were incubated twice for 15 min in PBS containing 0.1% active DEPC and equilibrated for 15 min in 5× saline sodium citrate (SSC). Sections were prehybridized for 2 h at 68°C in a hybridization mix (50% formamide, 5× SSC, 500 μg/mL herring sperm DNA, and 250 μg/mL yeast tRNA). Sense and antisense *MMP2*, *MMP8*, and *MMP13* riboprobes labeled with digoxigenin (DIG)-UTP were denatured for 5 min at 80°C and added to the hybridization mix. Hybridization reactions were carried out overnight at 68°C. Prehybridization and hybridization reactions were performed in a box saturated with a 5× SSC and 50% formamide solution to avoid evaporation, and no coverslips were used. After hybridization, sections were washed for 30 min in 2× SSC at room temperature, 1 h in 2× SSC at 65°C, and 1 h in 0.1× SSC at 65°C. Probes bound to the section were detected immunologically using sheep anti-DIG Fab fragments covalently coupled to alkaline phosphatase and nitro blue tetrazolium chloride/5-bromo-4-chloro-3-indolyl phosphate (toluidine salt) as chromogenic substrate, according to the manufacturer’s protocol (Roche, Basel, Switzerland). Images were captured using an Eclipse TE2000-U microscope (Nikon, Seoul, Korea) and processed with Adobe Photoshop CS6 software (Adobe Systems, Seattle, WA, USA).

### Gelatin zymography

Gelatinase activity in endometrial tissue homogenates was determined by gelatin zymography. Endometrial tissues were homogenized in lysis buffer (1% [v/v] Triton X-100, 0.5% [v/v] Nonidet P-40, 50 mM NaCl, 10 mM Tris, 1 mM EDTA, 0.2 mM Na_3_VO_3_, 0.2 M phenylmethylsulphonyl fluoride, 0.5 μg/mL NaF, and a proteinase inhibitor cocktail [Roche, Switzerland]) at a ratio of 100 mg tissue per 1 mL buffer, and cellular debris was removed by centrifugation. Proteins (50 μg) were loaded in each lane and electrophoresed on 8% SDS-PAGE gels containing 0.3% gelatin under non-reducing conditions, followed by incubation overnight at 37°C in 50 mM Tris buffer (pH 7.5) with 1% (v/v) Triton X 100, 5 mM CaCl_2_, 1 μM ZnCl_2_, and 1 μM 4-aminophenylmercuric acetate. Gels were stained with Coomassie blue for 1 h and destained with tap water until bands could be clearly visualized. Gel images were captured using the Gel Doc EZ Gel Documentation System (Bio-Rad, Hercules, CA, USA) and processed with Adobe Photoshop CS6 software (Adobe Systems, USA).

### Explant cultures

Endometrial explant tissues were obtained from prepubertal gilts to determine the effects of ovarian steroid hormones or from gilts on day 12 of the estrous cycle to determine the effect of conceptus-derived cytokines on the expression of *MMP2*, *MMP8*, *MMP9*, *MMP12*, and *MMP13* mRNAs in the endometrium as described previously [23,24]. Endometrium was dissected from the myometrium and placed into warm phenol red-free Dulbecco’s modified Eagle’s medium/F-12 culture medium (DMEM/F-12; Sigma, USA) containing penicillin G (100 IU/mL) and streptomycin (0.1 mg/mL). Endometrial tissue was minced into small pieces using scalpel blades (2 to 3 mm^3^), and aliquots of 500 mg were placed into T25 flasks with serum-free modified DMEM/F-12 containing 10 μg/mL insulin (Sigma, USA), 10 ng/mL transferrin (Sigma, USA), and 10 ng/mL hydrocortisone (Sigma, USA). Endometrial explants were cultured immediately after mincing in the presence of 0, 5, 50, 500 pg/mL estradiol-17β (E2; Sigma, USA) or 0, 0.3, 3, 30 ng/mL progesterone (P4; Sigma, USA) or 0, 1, 10, or 100 ng/mL of IL-1β (IL1B) (Sigma, USA) or IFN-γ (IFNG) (R&D Systems, Minneapolis, MN, USA) in the presence of 30 ng/mL P4, 10 ng/mL E2, and 10 ng/mL IL1B in an atmosphere of 5% CO_2_ in air at 37°C. Doses were chosen to encompass the full range of physiological levels of E2, P4, IL1B, and IFNG [25]. Explant tissues were then harvested, and total RNA was extracted for real-time RT-PCR to determine the expression of *MMP2*, *MMP8*, *MMP9*, *MMP12*, and *MMP13* mRNAs.

### Statistical analysis

Data from real-time RT-PCR to assess *MMP2*, *MMP8*, *MMP9*, *MMP12*, and *MMP13* expression during the estrous cycle and pregnancy were analyzed by ANOVA using the general linear models procedures of SAS (Cary, NC, USA). Data from real-time RT-PCR to assess the effects of day of the estrous cycle (days 0, 3, 6, 9, 12, 15, and 18) and pregnancy (days 10, 12, 15, 30, 60, 90, and 114) in the endometrium and chorioallantoic tissue (days 30, 60, 90, and 114) and the effect of E2, P4, IL1B, and IFNG on *MMP2*, *MMP8*, *MMP9*, *MMP12*, and *MMP13* expression were analyzed by least-squares regression analysis. Data are presented as means with standard error of the mean. Differences were considered significant if p<0.05.

## RESULTS

### Expression of *MMP2*, *MMP8*, *MMP9*, *MMP12*, and *MMP13* in the endometrium during the estrous cycle and pregnancy

**Figure 1:**
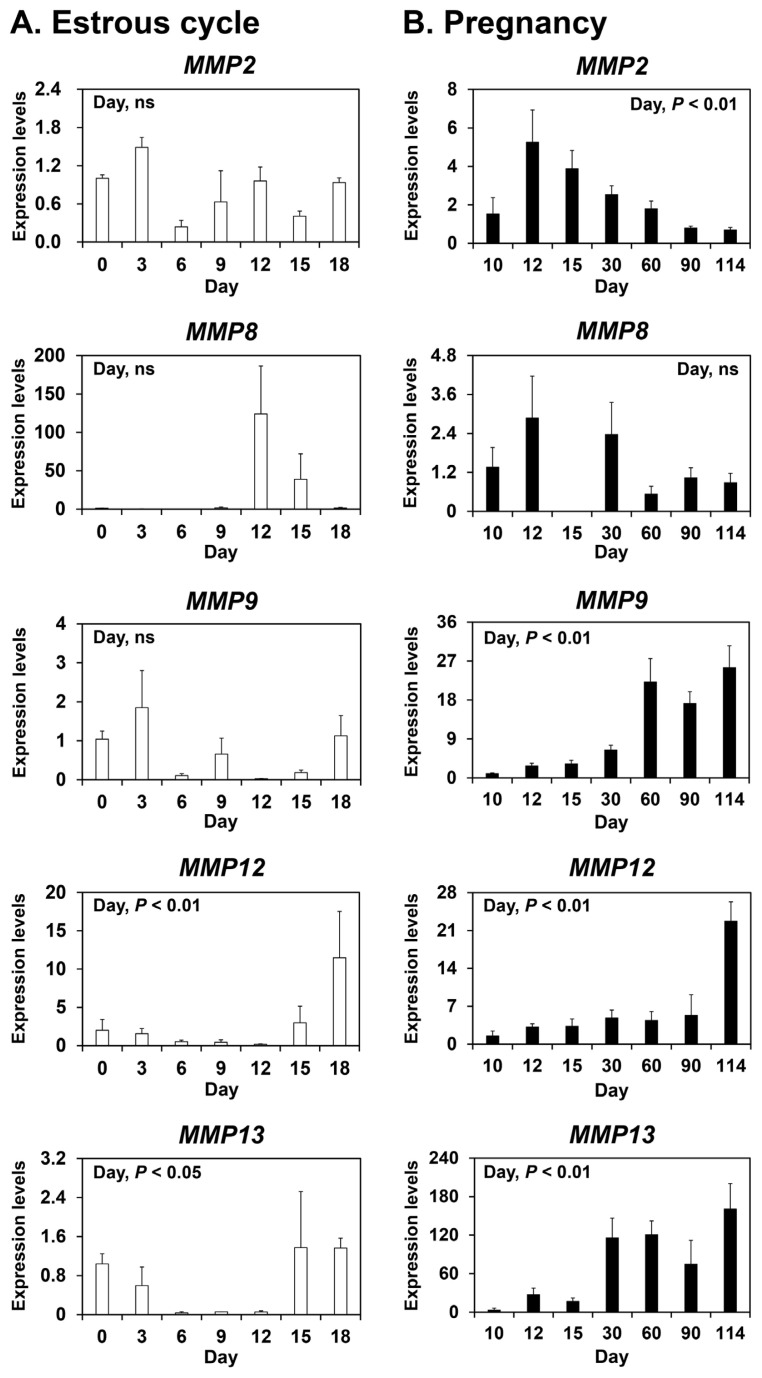
Expression of matrix metalloproteinase (*MMP*) 2, *MMP8*, *MMP9*, *MMP12*, and* MMP13* mRNAs in the endometrium during the estrous cycle (A) and pregnancy (B) in pigs. Endometrial tissue samples from cyclic and pregnant gilts were analyzed by real-time reverse transcription-polymerase chain reaction. Data are reported as expression relative to that detected on day 0 of the estrous cycle or day 10 of pregnancy after normalization of the transcript amount to the endogenous ribosomal protein L7, ubiquitin B, and TATA-binding protein controls. Data are presented as means with standard errors.

To determine whether *MMP2*, *MMP8*, *MMP9*, *MMP12*, and *MMP13* are expressed in the endometrium during the estrous cycle and pregnancy in pigs, we measured their relative abundance in the endometrium during the estrous cycle and pregnancy using real-time RT-PCR analysis (Figure 1). During the estrous cycle, the abundance of *MMP12* and *MMP13* mRNAs, but not *MMP2*, *MMP8*, and *MMP9* mRNAs, changed in the endometrium with the highest levels observed on day 18 (cubic effect of the day; p<0.01 for *MMP12*, p<0.05 for *MMP13*) (Figure 1A). During pregnancy, the abundance of* MMP2*, *MMP9*, *MMP12*, and* MMP13* mRNAs, but not *MMP8* mRNA, changed in the endometrium (linear effect of the day; p<0.01) (Figure 1B).

### Expression of *MMP2*, *MMP8*, *MMP9*, *MMP12*, and *MMP13* in conceptus tissues during early pregnancy and chorioallantoic tissues during later stage pregnancy

**Figure 2:**
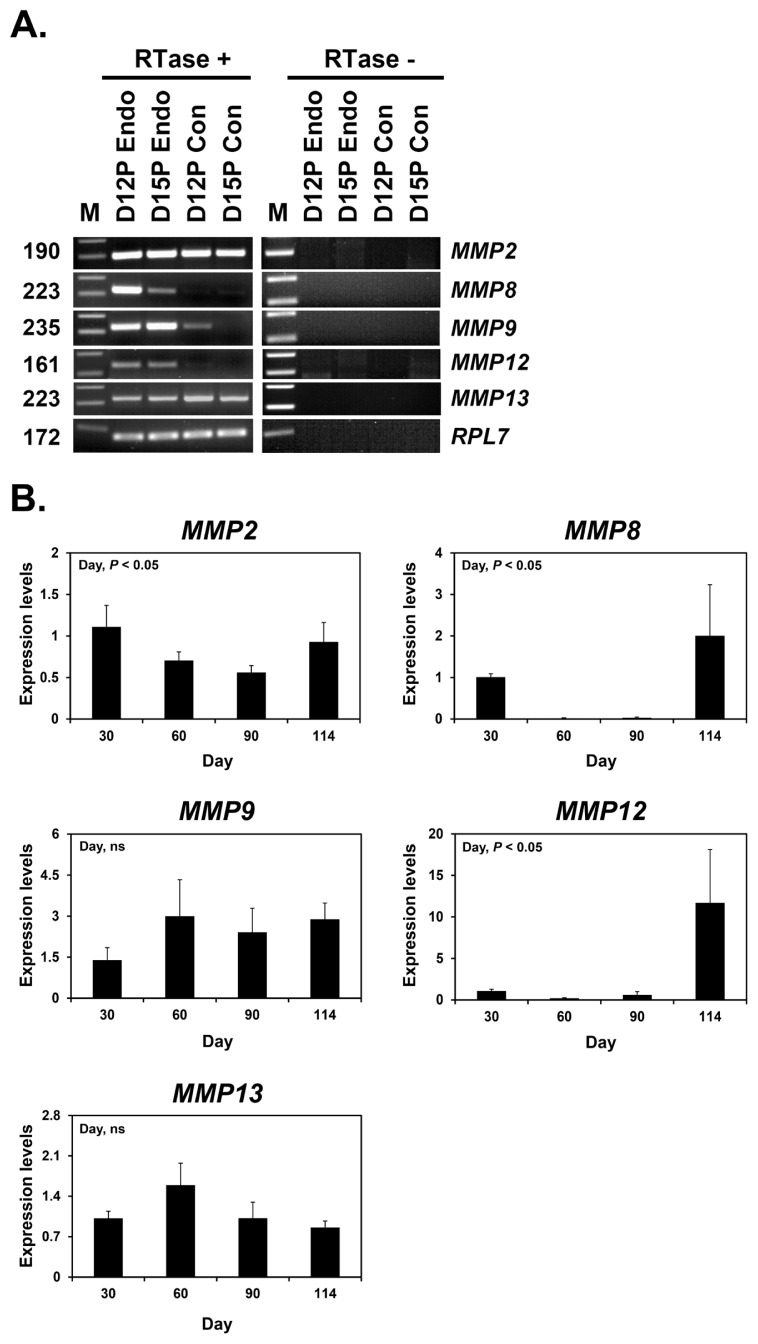
Expression of matrix metalloproteinase (*MMP*) 2, *MMP8*, *MMP9*, *MMP12*, and *MMP13* in conceptuses from days 12 and 15 of pregnancy (A) and chorioallantoic tissues during later pregnancy (B). A. Reverse transcription-polymerase chain reaction (RT-PCR) of *MMP2*, *MMP8*, *MMP9*, *MMP12*, and *MMP13* mRNA from pregnancy days 12 and 15 conceptuses using total RNA. Ribosomal protein L7 (*RPL7*) was used as a loading control. RTase +/−, with (+) or without (−) reverse transcriptase; M, molecular marker; D12P Endo, endometrium on day 12 of pregnancy; D15P Endo, endometrium on day 15 of pregnancy; D12 Con, day 12 conceptus; D15 Con, day 15 conceptus. B. Real-time RT-PCR analysis of the expression of *MMP2*, *MMP8*,* MMP9*, *MMP12*, and *MMP13* mRNAs in chorioallantoic tissues on days 30, 60, 90, and 114 of pregnancy. Data are reported as expressions relative to that detected on day 30 of pregnancy after normalization of the transcript amount to the endogenous ribosomal protein L7, ubiquitin B, and TATA-binding protein controls, and are presented as means with standard errors.

Next, we determined whether conceptuses during the peri-implantation period of pregnancy express *MMP2*, *MMP8*, *MMP9*, *MMP12*, and* MMP13* by RT-PCR using cDNAs from conceptuses from days 12 and 15 of pregnancy. *MMP2* and *MMP13* mRNA expression was detected in conceptus tissues on both days of pregnancy, while *MMP9* mRNA was detected only on day 12 and *MMP8* and *MMP12* mRNAs were not detected (Figure 2A). We also performed real-time RT-PCR analysis to determine if the expression of *MMP2*, *MMP8*, *MMP9*, *MMP12*, and* MMP13* changed in chorioallantoic tissues from day 30 to term pregnancy. The expression of *MMP2*, *MMP8*, and *MMP12* mRNAs, but not *MMP9* and *MMP13* mRNAs, changed during pregnancy (linear effect of the day for *MMP2* and *MMP12*, cubic effect of the day for* MMP8*; p<0.05) (Figure 2B).

### Localization of *MMP2*, *MMP8*, and *MMP13* mRNAs in the endometrium during the estrous cycle and at the maternal-fetal interface during pregnancy

**Figure 3:**
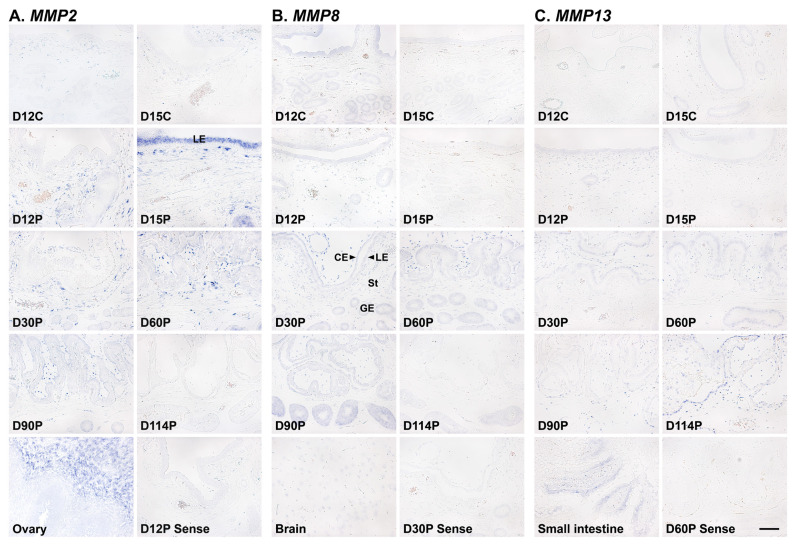
Localization of matrix metalloproteinase (*MMP*) 2 (A), *MMP8* (B),* MMP13* mRNA (C) by in situ hybridization in the endometrium during the estrous cycle and pregnancy in pigs. Representative uterine sections from days 12, 30, and 60 of pregnancy stained with sense RNA probes are shown as negative controls for *MMP2*, *MMP8*, and* MMP13*, respectively. Tissue sections from the ovary, brain, and small intestine are shown as positive controls for *MMP2*,* MMP8*, and *MMP13* mRNAs, respectively. D, day; C, estrous cycle; P, pregnancy; LE, luminal epithelium; GE, glandular epithelium; CE, chorionic epithelium; St, stroma. Bars = 100 μm.

To determine which cell type(s) express *MMP2*, *MMP8*, and *MMP13* mRNAs in the endometrium, we performed *in situ* hybridization analysis (Figure 3). *MMP2* mRNA was localized to stromal cells in the endometrium during the estrous cycle and pregnancy, and interestingly, luminal epithelial (LE) cell-specific *MMP2* expression was also detected on day 15 of pregnancy. In addition, *MMP2* expression was primarily localized to stromal cells in chorioallantoic tissue during pregnancy (Figure 3A). *MMP8* and *MMP13* expression was localized to epithelial and stromal cells in the endometrium during the estrous cycle and pregnancy, and also to epithelial and stromal cells in chorioallantoic tissue during pregnancy (Figures 3B and 3C).

### Presence of active MMP2 protein in endometrial tissues during the estrous cycle and early pregnancy

**Figure 4:**
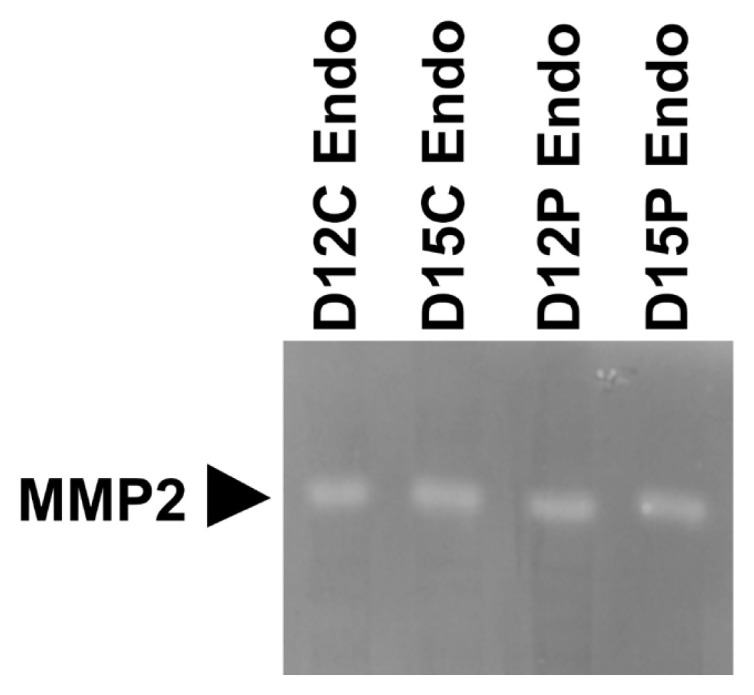
Gelatin zymography of matrix metalloproteinase (MMP) 2 in endometrial tissues on days 12 and 15 of the estrous cycle and pregnancy. Tissue homogenates were loaded into 8% sodium dodecyl sulfate-polyacrylamide gel electrophoresis gels containing 0.3% gelatin and electrophoresed under non-reducing conditions. After incubation overnight, gels were stained with Coomassie blue and destained with tap water. Unstained bands were detected with a molecular weight of approximately 75 kDa. D, day; C, estrous cycle; P, pregnancy; Endo, endometrium.

Having determined that MMPs were expressed in the endometrium during the estrous cycle and pregnancy, we further determined if MMP2 protein with enzymatic activity was present in the endometrium on days 12 and 15 of the estrous cycle and pregnancy using gelatin zymography (Figure 4). Clear single band of MMP with gelatinase activity and a molecular weight of approximately 75 kDa, which corresponds to pro-MMP2 [26], was detected in each lane.

### Effects of steroid hormones E2 and P4 and cytokines IL1B and IFNG on *MMP2*, *MMP8*, *MMP9*, *MMP12*, and *MMP13* expression in endometrial tissues

**Figure 5:**
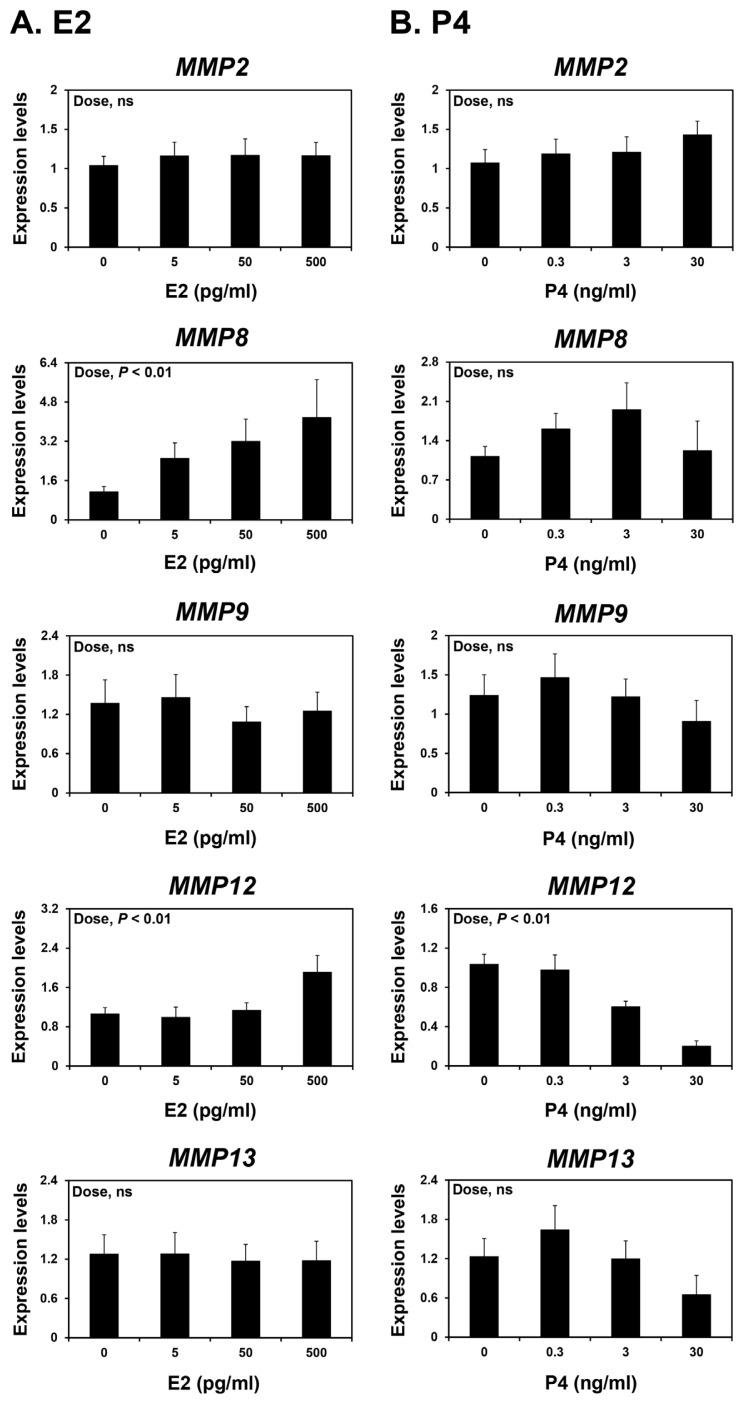
Effects of E2 (A) or P4 (B) on the expression of matrix metalloproteinase (*MMP*) 2, *MMP8*, *MMP9*, *MMP12*, and *MMP13* mRNAs in endometrial explant cultures. Endometrial explants from prepubertal gilts were cultured with 0, 5, 50, 500 pg/mL estradiol-17β (E2) or 0, 0.3, 3, 30 ng/mL progesterone (P4). The expression of mRNA was determined by real-time reverse transcription-polymerase chain reaction and expressed relative to that of *MMP2*, *MMP8*, *MMP9*, *MMP12*, and *MMP13* mRNA expression in the endometrial explants of the control group (0 ng/mL E2 or P4) after normalization of transcript amounts to endogenous ribosomal protein L7, ubiquitin B, and TATA-binding protein mRNAs. Data are presented as means with standard errors. These treatments were performed in triplicate using tissues obtained from each of three gilts.

Because the endometrium is a major target tissue of the steroid hormones E2 and P4 [25] and the expression of some *MMPs* varied during the estrous cycle and pregnancy, we investigated whether E2 and P4 affected the expression of *MMP2*, *MMP8*,* MMP9*, *MMP12*, and *MMP13* in endometrial tissues. To assess the individual effects of E2 or P4 and to exclude the possibility that these hormones influenced endometrial tissues during the estrous cycle, we utilized endometrial tissues from prepubertal gilts that had not yet undergone an estrous cycle. When we cultured endometrial explant tissues with increasing doses of E2 or P4, we found that E2 increased the expression of *MMP8* and *MMP12* (linear effect of dose; p<0.01) (Figure 5A), and P4 decreased the expression of *MMP12* (linear effect of dose; p<0.01) (Figure 5B). The expression of *MMP2*, *MMP9*, and *MMP13* in endometrial tissues was not affected by E2 or P4 treatment.

**Figure 6:**
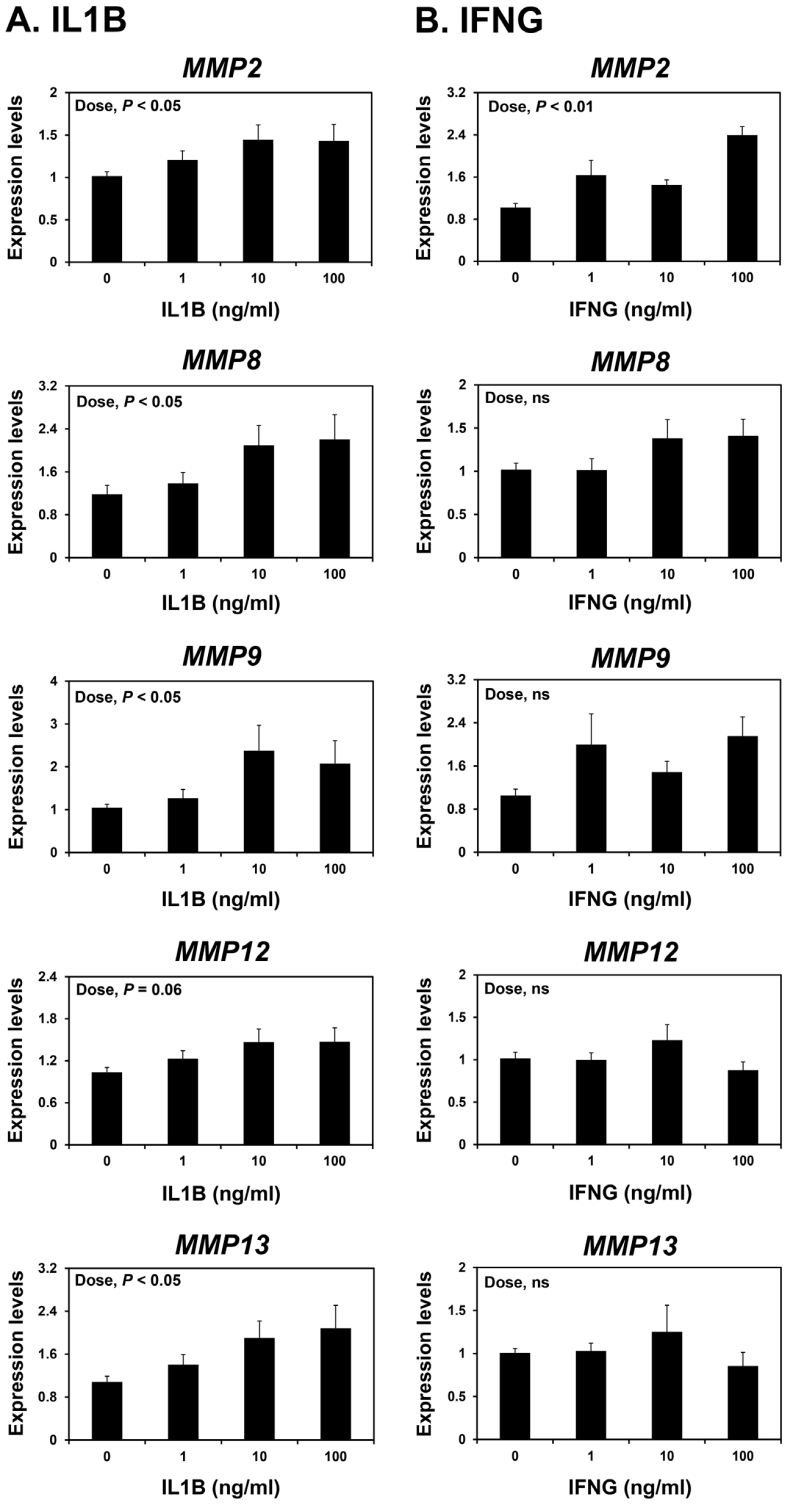
Effects of interleukin-1β (IL1B) (A) and interferon-γ (IFNG) (B) on the expression of matrix metalloproteinase (*MMP*) *2*, *MMP8*, *MMP9*, *MMP12*, and *MMP13* mRNAs in endometrial explant cultures. Endometrial explants from gilts on day 12 of the estrous cycle were cultured with 0, 1, 10, or 100 ng/mL IL1B or IFNG. The abundance of mRNA expression determined by real-time reverse transcription-polymerase chain reaction is reported relative to that for *MMP2*, *MMP8*, *MMP9*, *MMP12*, and *MMP13* mRNAs in endometrial explants of the control group (0 ng/mL IL1B or IFNG) after normalization of transcript amounts to endogenous ribosomal protein L7, ubiquitin B, and TATA-binding protein mRNAs. Data are presented as means with standard errors. These treatments were performed in triplicate using tissues obtained from each of three gilts.

Because the expression of *MMP2* in the endometrium were greatest at the implantation period, which corresponds to the time when the implanting conceptus secretes a significant amount of IL1B and IFNs [3,25], we postulated that IL1B and/or IFNG may affect the expression of MMPs in the endometrium during early pregnancy. We treated endometrial explant tissues from day 12 of the estrous cycle with increasing doses of IL1B or IFNG. IL1B increased the expression of *MMP2*, *MMP8*, *MMP9*, and *MMP13*, but not *MMP12*, in a dose-dependent manner (linear effect of dose; p<0.05) (Figure 6A), while IFNG increased the expression of *MMP2*, but not others, in endometrial explant tissues (linear effect of dose; p<0.01) (Figure 6B).

## DISCUSSION

The novel findings of this study in pigs are as follows: i) *MMP2*, *MMP8*, *MMP9*, *MMP12*, and *MMP13* are expressed in the pig endometrium in a stage-dependent manner; ii) conceptuses express *MMP2*, *MMP9*, and *MMP13* on day 12 of pregnancy and *MMP2* and *MMP13* on day 15 of pregnancy, and chorioallantoic tissues from day 30 to term pregnancy express *MMP2*, *MMP8*, *MMP9*, *MMP12*, and *MMP13* with differential expression patterns; iii) the localization of* MMP2*, *MMP8*, and *MMP13* expression in the endometrium is cell type-specific; iv) enzymatically active MMP2 protein is present in endometrial tissues on days 12 and 15 of the estrous cycle and pregnancy; v) E2 increases the expression of *MMP8* and* MMP12* and P4 decreases the expression of *MMP12* in endometrial explant tissues; and vi) IL1B increases the expression of *MMP2*, *MMP8*, *MMP9*, and *MMP13*, and IFNG increases the expression of *MMP2* in endometrial explant tissues.

MMPs are important regulators of tissue remodeling and play crucial roles in a variety of biological processes including apoptosis, angiogenesis, tissue repair, immune responses, and reproduction [5,6,8]. Endometrial expression of MMPs has been reported in several species, including humans, rodents, and cows [6,27–29]. In particular, MMPs play critical roles in regulation of endometrial tissue remodeling in primates and rodents in which dramatic endometrial tissue remodeling occurs during the reproductive cycle to maintain reproductive cyclicity and during pregnancy to form a hemochorial-type placenta [6,27,28]. Dysregulation of endometrial MMP expression is associated with infertility and early pregnancy loss in humans [27,28]. Some MMPs in rats are involved in trophoblast invasion and decidual remodeling during the implantation period [6]. In pigs, microscopic observations have shown that there are also some morphological changes in the endometrium during the estrous cycle [30]. Furthermore, several MMPs, including *MMP2*, *MMP8*, *MMP12*, and *MMP13* were found to be expressed in the endometrium during early pregnancy by transcriptome analysis [19,20]. However, the expression of MMPs in the endometrium throughout the estrous cycle and at the maternal-conceptus interface during pregnancy has not been fully elucidated. The present study in pigs clearly demonstrates that MMPs are expressed dynamically in the endometrium during the estrous cycle and pregnancy in a stage-specific manner and in the conceptus and chorioallantoic tissues during pregnancy. These results indicate that the expression of MMPs in the endometrium during the estrous cycle and at the maternal-conceptus interface during pregnancy is common among species regardless of implantation and/or placentation type.

We demonstrated that the expression of *MMP12* and *MMP13*, but not the other MMPs studied, changed during the estrous cycle with the greatest levels observed during the proestrus phase. This suggests that steroid hormones regulate the expression of MMPs, including *MMP12* and *MMP13*, in the endometrium during the estrous cycle. In explant cultures, E2 increased the expression of *MMP8* and *MMP12*, but not *MMP13*. By contrast, P4 decreased the expression of *MMP12* in endometrial explant tissues. These findings suggest that E2 and P4 of ovarian origin affect the expression of some endometrial MMPs, thereby contributing to endometrial tissue remodeling during the estrous cycle in pigs.

During pregnancy, the expression of *MMP2*, *MMP9*, *MMP12*, and *MMP13* in the endometrium and the expression of *MMP2*, *MMP8*, and *MMP12* in chorioallantoic tissue changed depending on the stage of pregnancy in this study. In the endometrium, the level of *MMP2* expression was greatest at the time of implantation and levels of *MMP9*, *MMP12*, and *MMP13* expression increased toward term pregnancy. In chorioallantoic tissues, *MMP2* expression was biphasic and *MMP8* and *MMP12* expression increased toward term pregnancy. The endometrium and placenta undergo dramatic tissue remodeling to provide an increased surface area for attachment between LE cells and the conceptus trophectoderm and reduce the interhaemal distance between the maternal and fetal circulatory systems [31]. As a result of extensive tissue remodeling at the maternal-fetal interface, a true epitheliochorial-type placenta is formed in pigs. Given that MMPs play important roles in tissue remodeling and MMPs were expressed stage-specifically at the maternal-conceptus interface during pregnancy in this study, MMPs expressed during pregnancy play a role in endometrial and placental tissue remodeling to form and maintain an epitheliochorial placenta. Indeed, we demonstrated that enzymatically active MMP protein, which corresponds to pro-MMP2 [26], was present in endometrial tissues from days 12 and 15 of the estrous cycle and pregnancy. In addition, increased expression of some MMPs in the endometrium and placenta toward term pregnancy suggests that these MMPs may be related to tissue remodeling at the maternal-fetal interface for the preparation of parturition.

Several cytokines, including ILs, IFNs, and TNF-α, induce the expression of MMPs in various cells and tissues [15]. During early pregnancy in pigs, implanting conceptuses produce cytokines IL-1β2 (IL1B2), IFN-γ (IFNG), and IFN-δ (IFND) [25], and cytokine production at the maternal-conceptus interface increases to induce parturition at term pregnancy [32]. In the current study, expression of *MMP2* was highest in the endometrium at the time of implantation, and the expression of *MMP9*, *MMP12*, and *MMP13* was greatest at term during pregnancy. Thus, we hypothesized that conceptus-derived cytokines might affect the endometrial expression of MMPs. Indeed, IL1B increased the expression of *MMP2*,* MMP8*, *MMP9*, and *MMP13*, and IFNG increased the expression of *MMP2* in endometrial explant cultures. It has also been proposed that other factors such as prostaglandin F2α, which is produced by the endometrium during the implantation period, induce the expression of endometrial MMP9 [33]. These data suggest that cytokines of maternal and conceptus origin induce endometrial MMP expression during pregnancy.

We detected MMP2, MMP8, and MMP9 mRNAs in epithelial and stromal cells in the endometrium and chorioallantoic tissues. In particular, MMP2 mRNA was primarily localized to stromal cells during the estrous cycle and pregnancy, but interestingly, it was also localized to LE cells on day 15 of pregnancy, which is the time when the implanting conceptus secretes a significant amount of IFNs in pigs [25], with the strongest signal intensity during pregnancy. In addition to their tissue remodeling actions, MMPs process bioactive molecules such as cytokines and chemokines [14]. MMP2 is known to cleave and release bioactive substrate molecules, including membrane-bound TNF family members, TNF superfamily 10 (TNFSF10), and CD40 ligand (CD40L) [34,35]. In pigs, TNFSF10 and CD40L are expressed by endometrial LE cells in response to conceptus-derived IFNG on day 15 of pregnancy and play an important role in immune regulation for the establishment of pregnancy [23,36]. The induction of *MMP2*, *TNFSF10*, and *CD40L* expression by IFNG in endometrial tissue and our finding that the highest expression of *MMP2* occurred on day 15 of pregnancy suggests that *MMP2* induced by conceptus-derived IFNG in the endometrium acts on the cleavage of TNFSF10 and CD40L in LE cells to activate TNFSF10- and CD40L-mediated immune responses during the implantation period in pigs. However, further studies are needed to clarify whether MMP2 cleaves TNFSF10 and CD40L and thereby is involved in immune regulation at the maternal-conceptus interface in pigs.

In conclusion, we demonstrated that MMPs were expressed in the endometrium of pigs during the estrous cycle and at the maternal-conceptus interface during pregnancy in a stage- and cell-type specific manner and that the steroid hormones E2 and P4 and cytokines IL1B and IFNG regulate the endometrial expression of MMPs. As key regulators of tissue remodeling and various bioactive molecules, MMPs expressed at the maternal-conceptus interface likely play important roles in the regulation of endometrial and placental tissue remodeling and activation of various cytokines and chemokines for the establishment and maintenance of pregnancy and the preparation of parturition in pigs.

## Figures and Tables

**Table 1:** Summary of primer sequences for RT-PCR, real-time RT-PCR, and *in situ* hybridization and expected product sizes

Primer	Sequence of forward (F) and reverse (R) primers (5′ → 3′)	Annealing temperature (°C)	Product size (bp)	GenBank accession no.
RT-PCR and real-time RT-PCR				
*MMP2*	F: ATGACGGAGAGGCTGACATC R: CCATACTTCACACGCACCAC	60	190	NM_214192.2
*MMP8*	F: TGCAACGATTCTTTGGACTG R: CAGAGTTGAAGGGCTTTTGC	60	223	XM_003129816.6
*MMP9*	F: TCGACGATGAAGAGTTGTGG R: TTACCGTCCCGAGTGAAGAG	60	235	NM_001038004.1
*MMP12*	F: CTGGACATGATGCACAAACC R: GCTTTCTGGATGGCGTAGTC	60	161	AM747278.1
*MMP13*	F: TGGCCATTCCTTAGGTCTTG R: TCCTCGGAGACTGGTAATGG	60	223	XM_003129808.5
*RPL7*	F: AAG CCA AGC ACT ATC ACA AGG AAT ACA R: TGC AAC ACC TTT CTG ACC TTT GG	60	172	NM_001113217
*UBB*	F: GCATTGTTGGCGGTTTCG R: AGACGCTGTGAAGCCAATCA	60	81	NM_001105309.1
*TBP*	F: AACAGTTCAGTAGTTATGAGCCAGA R: AGATGTTCTCAAACGCTTCG	60	262	DQ845178.1
*In situ* hybridization				
*MMP2*	F: ATGACGGAGAGGCTGACATC R: TCCAGTTAAAGGCAGCATCC	60	1,236	NM_214192.2
*MMP8*	F: TGCAACGATTCTTTGGACTG R: CAGAGTTGAAGGGCTTTTGC	60	223	XM_003129816.6
*MMP13*	F: TGGCCATTCCTTAGGTCTTG R: TCCTCGGAGACTGGTAATGG	60	223	XM_003129808.5

RT-PCR, reverse transcription polymerase chain reaction; *MMP*, matrix metalloproteinase; *RPL7*, ribosomal protein L7; *UBB*, ubiquitin B; *TBP*, TATA-binding protein.
